# Targeting of FGF-Signaling Re-Sensitizes Gastrointestinal Stromal Tumors (GIST) to Imatinib In Vitro and In Vivo

**DOI:** 10.3390/molecules23102643

**Published:** 2018-10-15

**Authors:** Sergei Boichuk, Aigul Galembikova, Pavel Dunaev, Ekaterina Micheeva, Elena Valeeva, Maria Novikova, Natalya Khromova, Pavel Kopnin

**Affiliations:** 1 Department of Pathology, Kazan State Medical University, Kazan 420012, Russia; ailuk000@mail.ru (A.G.); dunaevpavel@mail.ru (P.D.); miheeva.1973@bk.ru (E.M.); vevaleeva@ya.ru (E.V.); 2 N.N. Blokhin National Medical Research Center of Oncology, Moscow 115478, Russia; pretty_eve@mail.ru (M.N.); nkhromova@gmail.com (N.K.); pbkopnin@mail.ru (P.K.)

**Keywords:** gastrointestinal stromal tumor cells (GIST), imatinib (IM), resistance, FGFR signaling

## Abstract

Dysregulation of the fibroblast growth factor (FGF)/fibroblast growth factor receptor (FGFR) signaling pathway is frequently observed in multiple human malignancies, and thus, therapeutic strategies targeting FGFs and FGFRs in human cancer are being extensively explored. We observed the activation of the FGF/FGFR-signaling pathway in imatinib (IM)-resistant gastrointestinal stromal tumor (GIST) cells. Furthermore, we found that the activation of FGFR signaling has a significant impact on IM resistance in GISTs in vitro. Next, we tested the efficacy of BGJ398, a potent and selective FGFR1–3 inhibitor, in xenograft models of GISTs exhibiting secondary IM resistance due to receptor-tyrosine kinase (RTK) switch (loss of c-KIT/gain of FGFR2a). Five to eight-week-old female nu/nu mice were subcutaneously inoculated into the flank areas with GIST T-1R cells. Mice were randomized as control (untreated), IM, BGJ398, or a combination and treated orally for 12 days. IM had a moderate effect on tumor size, thus revealing GIST resistance to IM. Similarly, a minor regression in tumor size was observed in BGJ398-treated mice. Strikingly, a 90% decrease in tumor size was observed in mice treated with a combination of IM and BGJ398. Treatment with BGJ398 and IM also induced major histopathologic changes according to a previously defined histopathologic response score and resulted in massive myxoid degeneration. This was associated with increased intratumoral apoptosis as detected by immunohistochemical staining for cleaved caspase-3 on day 5 of the treatment. Furthermore, treatment with BGJ398 and IM significantly reduced the proliferative activity of tumor cells as measured by positivity for Ki-67 staining. In conclusion, inhibition of FGFR signaling substantially inhibited the growth of IM-resistant GISTs in vitro and showed potent antitumor activity in an IM-resistant GIST model via the inhibition of proliferation, tumor growth, and the induction of apoptosis, thereby suggesting that patients with advanced and metastatic GISTs exhibiting IM resistance might benefit from therapeutic inhibition of FGFR signaling.

## 1. Introduction

Fibroblast growth factors (FGFs) and their receptors (FGFR1–4) are ubiquitously expressed in human tissues and regulate a broad spectrum of physiologic processes including embryonic and tissue development, differentiation, proliferation, survival, migration, and angiogenesis. At the same time, dysregulation of FGF/FGFR signaling pathways is frequently observed in human cancer and has been documented for melanoma, hepatocellular carcinoma, breast, bladder, endometrial, head and neck, prostate, and lung cancers [1,2,3]. The molecular mechanisms of aberrant FGF-signaling in human cancers include the following: (i) FGFR-activating point mutations that promote dimerization or enhance kinase activity. For example, a point mutation in FGFR3 is detected in 50% to 60% of non-muscle, invasive urothelial carcinomas [4], whereas missense activating mutations of the extracellular domain in FGFR2 are documented in 12% to 14% of endometrial cancer cases [5,6]; (ii) FGFR gene amplification and overexpression, which triggers excessive FGFR-mediated signaling. For example, FGFR1 amplification has been found in up to 20% of squamous non-small cell lung carcinomas (NSCLC), 18% of osteosarcomas, 10% of breast carcinomas, and 6% of small cell lung carcinomas [7,8,9], and was associated with sensitivity to FGFR inhibitors in preclinical in vivo models. Similarly, FGFR2 amplification has been found in triple-negative breast and gastric cancers [10,11]. (iii) Inappropriate (excessive) FGFR-signaling might be also due to the presence of alternatively spliced FGFR variants with altered ligand selectivity [12]; (iv) aberrant autocrine/paracrine loops that result from an increased release of FGFs by tumor or stromal cells, thus promoting cancer cell survival, proliferation, and angiogenesis. This mechanism has been described for breast, prostate, and colorectal cancers, NSCLC, and hepatocellular carcinoma; (v) Finally, hyperactivation of FGFR–mediated pathways in cancer cells might be due to loss-of-function of negative regulators such as SPRY.

Important, aberrant FGFR signaling in human cancers very often has an impact on the disease prognosis and is usually associated with high sensitivity to FGFR inhibitors. For example, in hormone receptor-positive, HER2neu-positive, and triple-negative forms of breast cancer, FGFR1 amplification is associated with early relapse and poor outcomes [13,14] and also promotes the resistance of luminal B type breast cancer to endocrine therapy [15]. Similarly, FGFR2 amplification in breast and gastric cancers is associated with poorer prognosis and high sensitivity to FGFR inhibitors [16,17]. Similarly, selective sensitivity of FGFR2-mutant endometrial cancer to FGFR inhibitors was demonstrated in in vitro and in vivo models [18,19].

Despite these facts, very little is currently known about the impact of FGFR-signaling on GIST pathogenesis, prognosis, and sensitivity to targeted-based therapy and chemotherapy. Li Y. and co-authors demonstrated that FGF2 and FGFR1 are overexpressed in primary GISTs, suggesting that FGFR signaling may have an impact on IM efficacy [20]. Indeed, BGJ398, a selective small-molecule inhibitor of FGFR1–3, increased the growth inhibitory effect of IM in both of the IM-sensitive GIST lines (GIST T-1 and 882) used in this study. Moreover, a similar effect was observed in patient-derived GIST xenografts. Importantly, prolonged exposure (for 7 days) of GIST cells to IM induced reactivation of ERK signaling was repressed by the FGFR inhibition. Thus, IM-based therapy in GIST patients might trigger a feedback activation of FGFR signaling that can attenuate the antitumor effects of IM [20]. Our recent data also illustrates the significance of FGFR-signaling in GIST pathogenesis and therapy. For example, we showed that IM-resistant GIST cell lines established in our lab harbored a receptor-tyrosine kinase (RTK) switch (loss of c-KIT/gain of FGFR2), thus revealing the possibility of FGFR activation after prolonged IM exposure [21]. Moreover, we observed that aberrant FGFR signaling in IM-resistant GIST cells has an impact on GIST resistance to IM and topoisomerase II inhibitors [21,22]. The latter might be due to the ability of FGFR signaling to maintain effective repair of DNA double-strand breaks (DSBs) via a homology-mediated pathway [22].

Our present data provide evidence that FGFR signaling is activated in GISTs over the development of IM resistance. Importantly, we also observe that the inhibition of FGFR signaling in IM-resistant GIST restores tumor cell sensitivity to IM in vitro and in vivo. Indeed, we observe a substantial inhibition of cell growth in IM-resistant GIST (e.g., GIST T-1R) cells exposed to IM and BGJ398, a selective FGFR1–3 inhibitor. In contrast, no growth inhibition is observed when IM-sensitive GIST T-1 cells are treated IM and BGJ398, thus highlighting a potent FGFR-inhibitory effect that is specific for IM-resistant GISTs. Moreover, the inhibition of FGFR signaling effectively potentiates the antitumor activity of IM in an IM-resistant GIST xenograft model via the inhibition of proliferation, tumor growth, and the induction of apoptosis, thereby suggesting that patients with advanced and metastatic GISTs exhibiting IM resistance might benefit from the therapeutic inhibition of FGFR signaling.

## 2. Results

### 2.1. FGF/FGFR Signaling Is Activated in IM-Resistant GIST Cells

We showed recently that inhibition of the FGF/FGFR signaling pathway by BGJ398, a selective pan-FGFR inhibitor, re-sensitized IM-resistant GIST cells (GIST T-1R) to IM. Of note, no synergistic effect of FGFR- and c-KIT inhibition was observed in the parental IM-naïve GIST T-1 cell line. This data suggests that the activation of FGF/FGFR signaling might be responsible for IM resistance in GISTs [21]. To examine whether FGFR signaling is activated in IM-resistant GISTs, we performed the phospho-RTK array assay and compared the expression of phosphorylated RTKs in IM-naïve vs. resistant GIST T-1 cells. We found that IM-resistant GIST cells exhibited a coordinated activation of RTKs involved in FGFR-signaling pathways—RAS-MAPK and PI3K-AKT.

**Figure 1:**
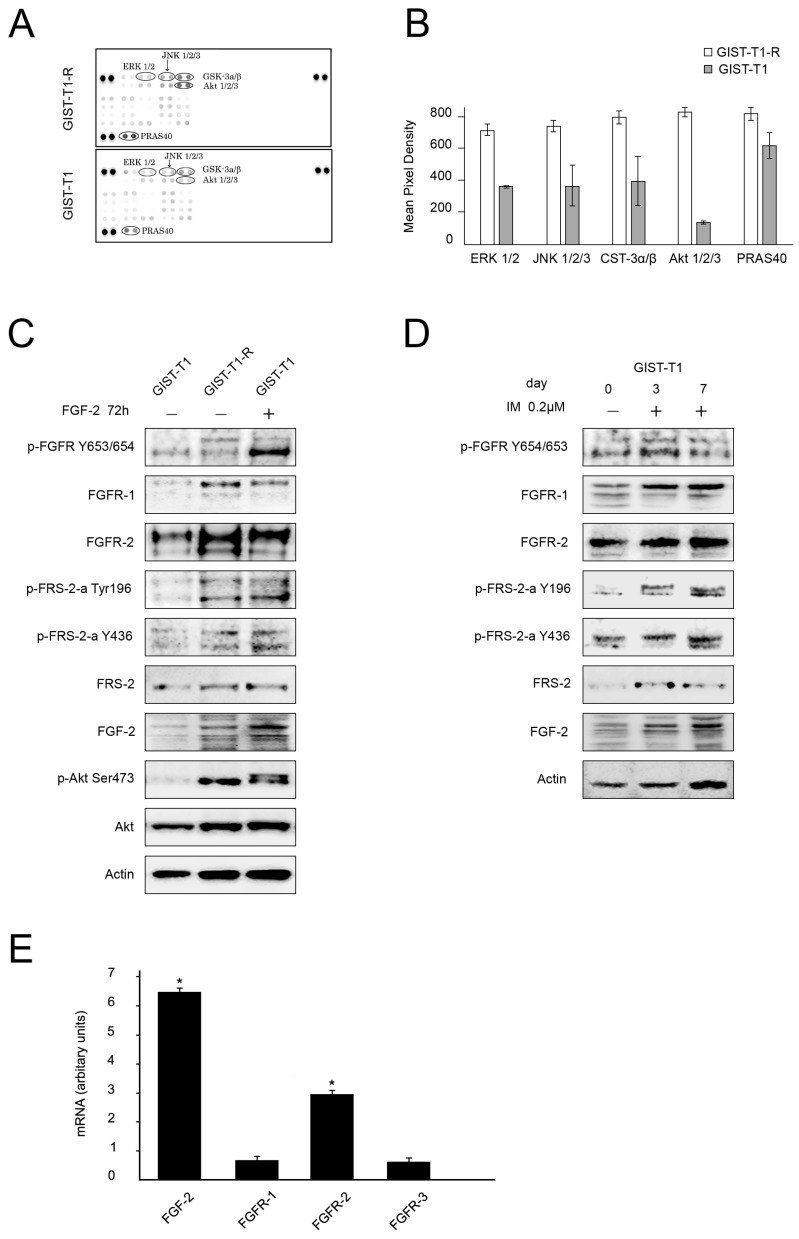
Phospho-kinase profiling of IM-resistant and sensitive GIST T-1 cells. (**A**) Total cell lysates (300 µg) from GIST T-1 (lower panel) and GIST T-1R cells (upper panel) were analyzed by phospho-kinase array. Each TK was spotted in duplicate; the spots at each corner are positive controls. (**B**) Quantification by mean pixel density revealed that three phosphorylated proteins are dysregulated in GIST T-1R when compared to parental GIST T-1 cells. (**C**) Immunoblot analysis of total and phosphorylated forms of fibroblast growth factor receptor (FGFR), AKT, FRS-2, FGF-2 in IM sensitive (GIST T-1) and resistant (T1-R). Actin stain was used as a loading control. GIST T-1 treated with FGF-2 for 72 h was used as a positive control. (**D**) Immunoblot analysis of the total and phosphorylated forms of FGFR, FRS-2, and FGF-2 in non-treated (−) and IM-treated (+) GIST T-1 cells. (**E**) Changes in the expression level of *FGFR1–3* and *FGF2* genes in GIST T-1R cells relative to GIST T-1 cells, as determined by reverse transcription quantitative polymerase chain reaction. Glyceraldehyde-3-phosphate dehydrogenase (GAPDH) was amplified as an internal control. Data are expressed as the mean ± SD of three independent experiments. Statistical analyses of parental and T1-R-cells were performed by using Student’s *t*-tests, * *p* ≤ 0.05.

This was evidenced by an increased expression of the phosphorylated forms of AKT1/2/3 and ERK1/2 (Figure 1A,B). Moreover, the phosphorylation of several distinct MAPK kinases, including JNK that acts downstream of FGFR signaling, was also increased in IM-resistant GIST cells. Similarly, the phosphorylation of GSK3β, a multifunctional serine/threonine kinase, known as a critical element of the PI3K/AKT and PKC pathways and a promoter of cell growth and survival, substantially increased in IM-resistant GIST cells. In addition, expression of the proline-rich protein (PRAS40) that binds to 14-3-3 proteins and to mTOR to transduce AKT signaling to the mTOR complex was much higher in IM-resistant GIST cells when compared to parental IM-sensitive GIST T-1 cells.

Phospho-kinase array data is corroborated by immunoblot analyses that revealed an increased expression of pAKT S473 in IM-resistant GIST T-1R cells when compared with parental IM-sensitive GIST T-1 cells (Figure 1C). Activation of FGFR signaling in IM-resistant GIST T-1R cells was also evidenced by an increased expression of Tyr-phosphorylated FGF receptor (pFGFR Y653/654) and Tyr-phosphorylated FGFR substrate 2 (FRS2 Tyr196) which is a downstream substrate of FGFR signaling (Figure 1C). Interestingly, the expression of FGF-2 in IM-resistant T-1 cells was also at a higher level when compared to the parental IM-sensitive T-1 cells. Interestingly, the activation of FGFR signaling in GIST cells was also observed even after a short-term culture with IM. Indeed, the incubation of IM-sensitive GIST T-1 cells with a low dose of IM (0.2 μM) over 7 days increased the expression of FGFR1, phospho-FRS2 and FGF-2 in a time-dependent manner (Figure 1D).

To examine whether the components of the FGF/FGFR signaling pathway become up-regulated at the transcriptional level in IM-resistant GISTs, we compared the mRNA expression of FGF-2 and FGFR1–3 in GIST T-1 vs. T-1R cells. We observed a 6.5-fold increase in FGF-2 mRNA and a 2.5-fold increase in FGFR2 mRNA in IM-resistant GIST T-1 cells when compared to IM-sensitive parental GIST T-1 cells (Figure 1E). This is in agreement with the Western blotting data shown in Figure 1C.

The role of the autocrine-mediated pathway in FGF/FGFR-signaling in IM-resistant GISTs was examined by measuring the concentration of FGF-2 in GIST supernatant. We observed a ~3-fold increase of FGF-2 in IM-resistant GIST T-1R cells when compared with IM-naïve parental GIST T-1 cells. Moreover, the level of FGF-2 in GIST T-1 supernatant increased on days 3 and 7 after IM treatment (data is not shown), thus suggesting the activation of the autocrine FGF/FGFR signaling loop in IM-treated GISTs which might attenuate the therapeutic (e.g., pro-apoptotic and anti-proliferative) effects of IM-based therapy.

### 2.2. BGJ398 Restores the Growth Inhibitory Effect of IM in GIST T1-R Cells In Vitro

**Figure 2:**
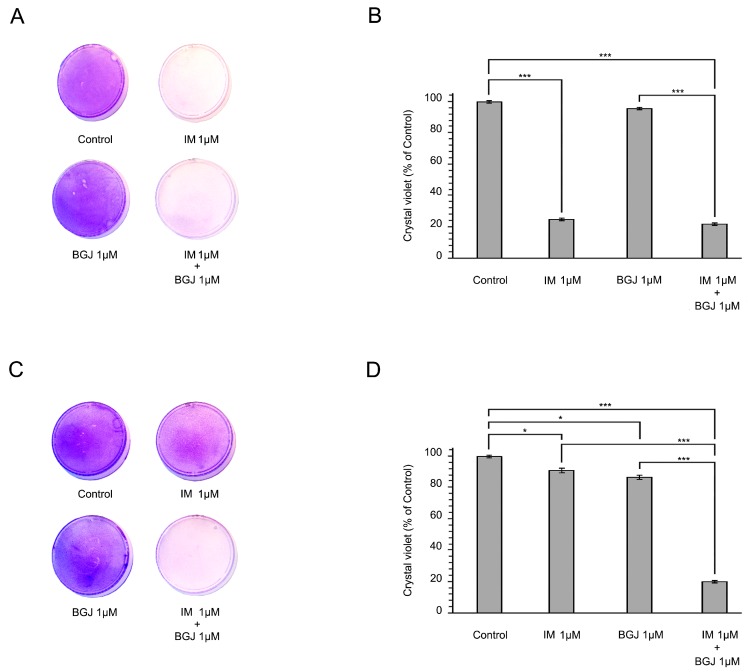
Inhibition of fibroblast growth factor receptor (FGFR) signaling sensitizes IM-resistant GIST cells to IM. Crystal violet staining of GIST T-1 (**A**) and GIST T-1R (**C**) cells. Cells were treated with DMSO (control), BGJ398 (1 μmol/L), or IM (1 μmol/L) alone or in combination for 6 days. The culture dishes were fixed, stained with crystal violet, and photographed. (**B**,**D**) Quantitative analysis of crystal violet staining of IM-sensitive (**B**) and IM-resistant GIST cells (**D**). The plates were dried, crystal violet was dissolved using 0.1% SDS solution, and absorbance was measured at 590 nm. The graphs represent the mean ± SD. * *p* < 0.05; *** *p* < 0.001.

To further examine the impact of FGFR inhibition on the anti-proliferative capacity of IM in IM-naïve vs. resistant GIST T-1 cells, we utilized the long-term viability assay and crystal violet staining procedure. The cells were exposed to IM or BGJ398 (a selective FGFR1–3 inhibitor) alone or in combination for 6 days and then further stained with crystal violet according to the protocol. As expected, IM alone substantially inhibited the proliferation of IM-naïve GIST T-1 cells, whereas no inhibitory effect was found in IM-resistant GISTs (Figure 2A,C, respectively). FGFR inhibition was associated with minor anti-proliferation activities on IM-resistant and naïve GIST cells as well. Strikingly, we observed a substantial inhibition in cell growth in IM-resistant GIST (e.g., GIST T-1R) cells exposed to IM and BGJ398 (Figure 2C,D). No increase in GIST T-1 cell growth inhibition was observed when IM-treated cells were compared to the cells exposed to IM and BGJ398 (Figure 2B), thus highlighting a potent FGFR-inhibitory effect that is specific for IM-resistant GISTs.

### 2.3. Inhibition of the RAF/MAPK-Signaling Pathway Restores the Sensitivity of IM-Resistant GIST Cells to IM

**Figure 3:**
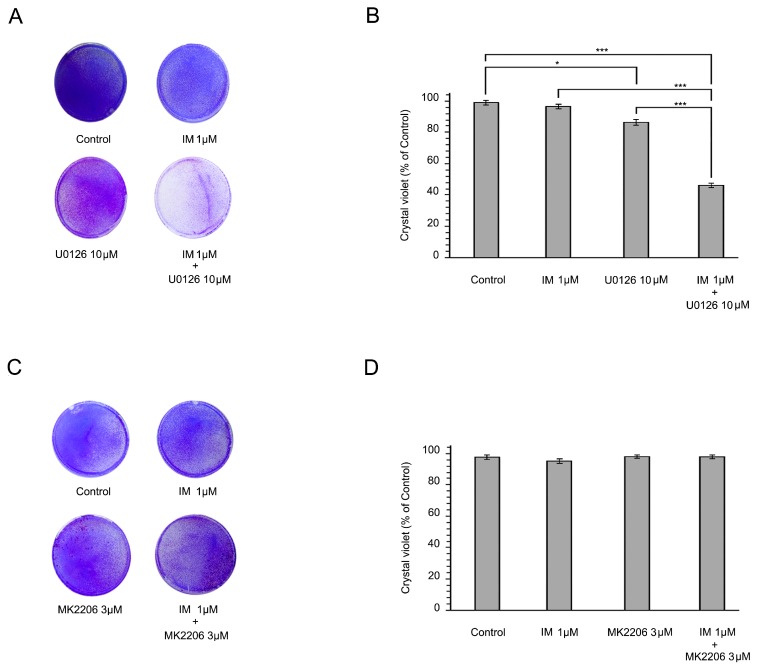
Influence of MEK and AKT inhibition on GIST T-1R resistance to IM. Crystal violet staining of GIST T-1R cells (**A**,**C**). Cells were treated with DMSO (control), IM (1 μmol/L), a MEK inhibitor (U0126; 1 μmol/L) or an AKT inhibitor (MK2206; 1 μmol/L) alone or in combination for 6 days. The culture dishes were fixed, stained with crystal violet, and photographed. (**B**,**D**) Quantitative analysis of crystal violet staining of GIST cells treated as described above. The plates were dried, crystal violet was dissolved using 0.1% SDS solution, and absorbance was measured at 590 nm. The graphs represent the mean ± SD. * *p* < 0.05; *** *p* < 0.001.

Given that the RAF/MAPK pathway is one of the intracellular downstream signaling cascades of FGF/FGFR signaling, we decided to examine whether the inhibition of the MEK/ERK pathway re-sensitizes IM-resistant GISTs to IM. For this purpose, GIST T-1R cells were treated with a potent MEK/ERK inhibitor U0126 (10 µM) alone or in combination with IM for 6 days and a long-term viability assay and crystal violet staining were performed. We found that MEK/ERK inhibition reversed the sensitivity of GIST T-1R cells to IM which was evidenced by significant inhibition of cell growth in GIST T-1R cells exposed to the combination of IM and U0126 (Figure 3A,B). This effect was not due to the inhibition of the KIT-mediated pathway, as this type of IM-resistant GIST cells exhibits an RTK switch (loss of c-KIT/gain of FGFR2a), as described above. Of note, U0126 alone has minor anti-proliferation activity on IM-resistant GIST cells (Figure 3A,B). In contrast to U0126, MK2206 (3 µM), a selective AKT1/2/3-inhibitor, failed to re-sensitize GIST-T1R cells to IM (Figure 3C,D).

Taken together, this data illustrates that the MAPK signaling pathway plays an important role in IM resistance in GIST T-1R cells.

### 2.4. Inhibition of FGFR Signaling Potentiates IM-Induced Growth Inhibition of GISTs In Vivo

**Figure 4:**
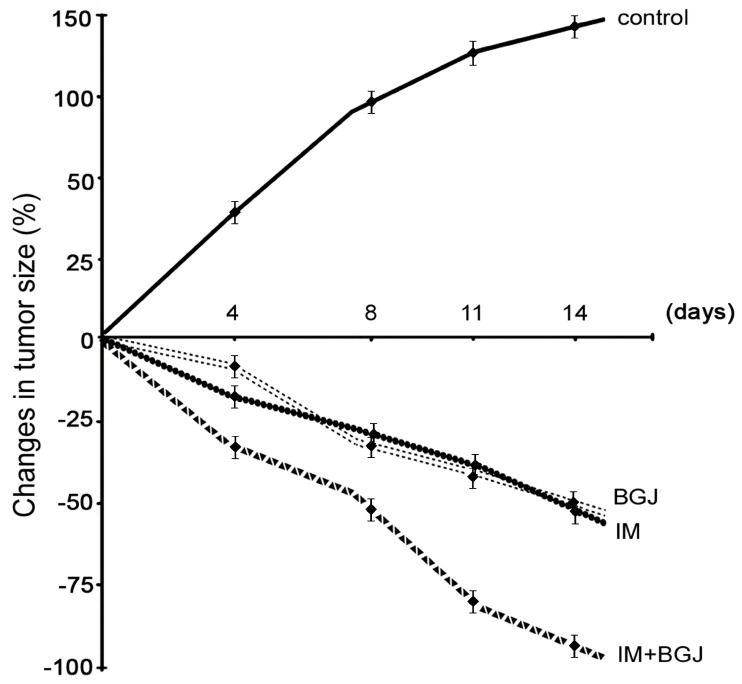
Dynamics of the tumor volume of GIST T-1R xenografts. Nude mice bearing GIST T-1R xenografts were orally administered daily with 50 μL of either a vehicle (negative control), IM (50 mg/kg), BGJ398 (20 mg/kg), or a combination of the drugs indicated above. The changes in tumor size were calculated as a percentage of the baseline.

Given the improved efficiency of combined FGFR and KIT inhibition in IM-resistant GIST cells in vitro, we further tested the antitumor activities of IM and BGJ398 in vivo using an IM-resistant GIST xenograft model. IM-resistant GIST cells were injected into the flanks of female adult athymic nude mice and tumors were allowed to grow for at least for 2 weeks. After 2 weeks, the animals were randomized into four groups, treated with a vehicle (control), IM (50 mg/kg), BGJ398 (20 mg/kg), or a combination for 14 days. As expected, vehicle-treated mice (control) demonstrated a continuous increase in tumor size from the baseline over the 2-week period (Figure 4). In IM-treated animals, tumors showed a moderate regression in size when compared to the baseline, thus revealing the resistance of GIST cells to IM. The in vivo administration of BGJ398 had a moderate impact on the tumor size. Thus, IM and BGJ398 had induced a regression in tumor size of up to 35% to 45% by the end of the experiment. Strikingly, a substantial (>90%) decrease in tumor size was observed in mice treated with a combination of IM and BGJ398 (Figure 4). Of note, no toxicity was observed in any animal groups over the whole experimental period (2 weeks).

**Figure 5:**
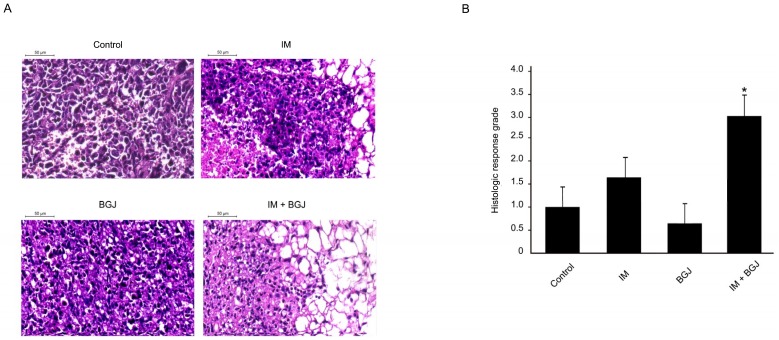
In vivo anti-tumor activity of BGJ38 and IM in a murine GIST xenograft model. (**A**) Histopathologic response (HR) of GIST T-1R xenografts to treatment with IM or BGJ398 used alone or in combination in comparison with a placebo (hematoxylin and eosin, ×10 magnification). (**B**) The data shown in graph represents the average of at least six tumors per group. Columns: mean + SE; * *p* ≤ 0.05.

Next, we assessed the morphological changes in GISTs treated with IM or BGJ398 alone or in combination. Histopathologic response (HR) grading scores (1–4) were used to assess the tumor response to the treatments indicated above. We found that IM alone induced local areas of central necrosis as assessed by HE staining (grade 1), whereas minimal signs of mixoid degeneration and fibrosis were observed in tumors treated with IM (Figure 5A).

In contrast, BGJ398, a selective pan-FGFR inhibitor had little impact on the histopathological parameters indicated above (Figure 5A) or on the HR score (Figure 5B). Indeed, minimal signs of fibrosis were detected in <20% of GISTs xenografts treated with BGJ398. Strikingly, mice bearing GIST xenografts treated with a combination of IM and BGJ398 exhibited a substantial mixoid degeneration of the tumors that reflected a maximal HR (grades 3 and 4) (Figure 5A,B).

### 2.5. BGJ398 Enhances the Pro-Apoptotic and Anti-Proliferative Activity of IM in GISTs In Vivo

**Figure 6:**
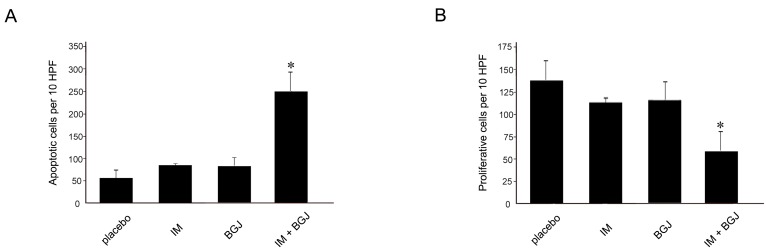
Pro-apoptotic activity and anti-proliferative activities of IM and BGJ398 in a murine GIST xenograft model. Quantification of the apoptotic cells visualized by immunohistochemical (IHC) staining for cleaved caspase-3 (**A**), or proliferative cells stained for Ki-67, a proliferative marker (**B**), in GIST xenografts treated with IM or BGJ398 alone or in combination in comparison with a placebo. At least six tumors per group were examined. Columns: mean + SE; * *p* ≤ 0.05.

To corroborate these findings, we examined the anti-proliferative and pro-apoptotic effects of IM and BGJ398 used alone or in combination. As expected, control (non-treated) tumors showed a high mitotic activity as detected by immunohistochemical (IHC) staining for Ki-67 (Figure 6A). IHC staining for cleaved caspase-3 indicated a low number of apoptotic cells (Figure 6B). IM modestly decreased the proliferative capacity of GISTs (Figure 6A) but had no significant impact on the number of apoptotic (e.g., caspase-3-positive) cells (Figure 6B). This fact revealed that the GIST T-1R cells used in the present study were IM resistant. Again, GIST xenografts treated with a combination of IM and BGJ398 exhibited a significant reduction in mitotic activity (Figure 6A). Similarly, a substantial increase in apoptotic cells was observed in GIST xenografts treated with IM and BGJ398 (Figure 6B).

Collectively, this data illustrates that the combined use of IM and BGJ398, a selective pan-FGFR inhibitor, re-sensitized IM-resistant GISTs to IM in vivo due to the inhibition of proliferation, tumor growth, and induction of apoptosis.

## 3. Discussion

Aberrant FGF/FGFR signaling is well-documented for multiple human malignancies (melanoma, hepatocellular carcinoma, breast, bladder, endometrial, head and neck, prostate, and lung cancers) and plays an important role in disease prognosis and sensitivity to FGFR inhibitors [16,17,18,19]. BGJ398 (Novartis), an orally bioavailable selective pan-FGFR kinase inhibitor, has shown anti-tumor activity in preclinical models as well as preliminary clinical activity against advanced solid tumors harboring FGFR alterations [23,24]. Inhibition of the FGFR-signaling pathway was also associated with clinical benefits for cancer patients who failed to respond to chemotherapy and targeted-based therapy. For example, the results of a phase II trial (NCT02150967) showed meaningful clinical activity of BGJ398 against advanced/metastatic and chemotherapy-refractory cholangiocarcinoma with FGFR2 fusions who progressed with first-line chemotherapy [25]. Importantly, the activation of FGF/FGFR signaling might also have an impact on the sensitivity of cancer cells to chemotherapeutic agents. For example, targeted FGFR signaling regulates the chemoresistance of head and neck cancer stem cells and sensitizes them to cisplatin [26].

A limited amount of data describing the role of FGFR signaling in GIST pathogenesis and therapy is currently available. Li Y. and co-authors demonstrated that FGF2 and FGFR1 are overexpressed in primary GISTs, suggesting that FGFR signaling may have an impact on IM efficacy. Important, IM treatment of GIST cells in vitro led to reactivation of ERK signaling, also suggesting that IM-based therapy in GIST patients might trigger a feedback activation of FGFR signaling that can further attenuate IM efficiency [20]. Activation of FGF/FGFR signaling in GISTs was suggested as one of the mechanisms responsible for IM resistance. Indeed, FGF2 expression increased in IM-resistant GIST cells in vitro and in tumor specimens from IM-resistant GIST patients. Moreover, RNAi-mediated silencing of FGFR3 in IM-resistant GIST cells inhibited their growth, and the combined inhibition of KIT and FGFR3 has a potent synergistic growth-inhibitory effect on IM-resistant GIST cells. This data, for the first time, highlights the importance of FGF/FGFR inhibition in overcoming IM resistance in GISTs, thus suggesting that targeting with FGFR3 might be considered as a promising strategy to improve the therapy of GIST patients with de novo or acquired resistance to IM [27].

These preclinical results illustrated a rationale for combining IM and FGFR inhibitors, such as BGJ398, in the therapy of GIST. A recent clinical study (https://clinicaltrials.gov/ct2/show/NCT02257541) evaluated the pan-FGFR inhibitor BGJ398 in combination with IM in advanced and metastatic GIST. The primary endpoint of this trial was to examine the safety of the combination therapy (IM and BGB 398), to define the maximum tolerated dose (MTD) and the recommended phase II dose and schedule. The secondary objectives of the trials included the investigation of the pharmacokinetics of BGJ398 and IM and the progression free survival (PFS) and clinical benefit rates (complete response (CR) + partial response (PR) + stable disease (SD)) after 32 weeks of combination therapy. Sixteen patients with locally advanced or metastatic GIST that had progressed on IM were enrolled in this study. Two patients experienced dose limiting toxicities from the combination therapy of BGJ398 and IM. No objective responses (CR and PR) were observed; however, stable disease was observed in seven patients (44%) and a clinical benefit rate of ≥32 weeks was observed in three of the 12 evaluable patients (25%). Unfortunately, due to withdrawal of sponsor support, the study was terminated earlier before the MTD with this dosing schedule was defined [28].

In previous studies published by our group, BGJ398, a potent and selective FGFR1–3 inhibitor, resulted in a prominent increase in apoptotic activity in IM-resistant GIST cells in vitro when used in combination with IM. In contrast, the use of BGJ398 and IM alone has no pro-apoptotic or anti-proliferative activities in IM-resistant GIST cells [21]. We also showed recently that the inhibition of FGFR-signaling in IM-resistant GIST cells sensitized them to the low doses of the topoisomerase II inhibitors, doxorubicin and etoposide. The potential molecular mechanisms mediating this effect were due to the substantial decrease of Rad51 recombinase expression and the attenuation of homology-mediated DNA repair [22].

Our current study provides the evidence that the FGF/FGFR signaling pathway is activated in IM-resistant GIST cells. Indeed, we found an increased expression of Tyr-phosphorylated FGF receptor (pFGFR Y653/654). It is well-known that activated FGFR phosphorylates adaptor proteins for four major intracellular signaling pathways: RAS-MAPK, PI3K-AKT, PLCγ, and signal transducer and activator of transcription (STAT) [29,30]. Activation of the RAS-MAPK and PI3K-AKT pathways is initiated by the phosphorylation of FRS2α. FRS2α phosphorylation and ERK1/2 activation are partially dependent on the phosphorylation of Y463 and the presence of CRKL. pY463 directly interacts with the adapter protein CRKL and with much lower affinity to the related protein CRK [31,32,33,34]. Downstream of RAS and PI3K, FGFR signaling has been shown to regulate several distinct MAP kinases including ERK1/2, JNK, and p38 [35,36,37]. Our current data illustrates an increased expression of Tyr-phosphorylated FGFR substrate 2 (FRS2 Tyr196) in GIST T-1R cells when compared to the parental IM-sensitive GIST T-1 cell line. GIST T-1R cells exhibited signs of activation of the downstream PI3K/AKT- and RAS/MAPK-mediated signaling pathways which was evidenced by an increased expression of the phosphorylated forms of the kinases (AKT, ERK, JNK and GSK) as well as PRAS40 which transduces AKT signaling to the mTOR complex. Of note, the changes indicated above were not due to the activation of KIT-mediated signaling pathway since the expression of pKIT Y719 was substantially decreased in this particular GIST T-1 subline. Important, we also showed here that IM time-dependently activates FGFR signaling in GISTs during short-term culture (3–7 days). This data is in agreement with previous findings that illustrated the activation of FGFR-signaling in IM-naive GIST cells cultured with low (0.2 µmol/L) doses of IM for up to 7 days [28]. Thus, IM activates the FGFR-signaling pathway in GISTs which might be associated with the development of the IM-resistant phenotype.

Therefore, inhibition of FGFR signaling might be beneficial for patients who have acquired resistance after IM-based therapy. Our present data illustrates that FGFR inhibition has a potent growth inhibitory effect in vitro on GIST cells exhibiting secondary IM resistance due to activation of the FGF/FGFR signaling pathway. This was also revealed in a xenograft model of GIST. Indeed, the growth of the xenografts was markedly attenuated in a group of mice treated with a combination of IM and BGJ398 when compared with control-treated animals and animals treated with BGJ398 or IM alone. The combined use of both RTK inhibitors indicated above led to a significant histopathological response according to a previously described histopathologic response score [38]. The morphological changes in the group of mice received that the combination therapy included the substantial decrease of the number of tumor cells and broad zones of myxoid degeneration.

Important, the combined therapy of BGJ398 and IM significantly increased intratumoral apoptosis as evidenced by immunohistochemical staining for cleaved caspase-3, whereas the number of mitotic cells, as measured by positivity for Ki-67, was reduced, thereby clearly demonstrating the effectiveness of the treatment.

Taken together, the inhibition of FGFR signaling in IM-resistant GISTs can re-sensitize them to IM, inhibit cancer cell proliferation, and induce apoptosis. Moreover, the combination of BGJ398 and IM treatment showed significant antitumor activity in vivo using a GIST xenograft mouse model.

Collectively, these data suggest that patients with advanced and metastatic GISTs exhibiting IM resistance due to RTK switch (loss of c-KIT/gain of FGFR) might benefit from the targeting of IM-resistant GIST cells by therapeutic inhibition of FGFR signaling.

## 4. Materials and Methods

### 4.1. Chemical Compounds

Imatinib (IM), MK2206 and BGJ 398 were purchased from SelleckChem (Houston, TX, USA). U0126 was purchased from Sigma (St. Louis, MO, USA).

### 4.2. Antibodies

The primary antibodies used for immunoblotting were as follows: phospho-KIT Y719, phospho-AKT S473, АКТ, phospho-FRS2α Y196 and Y436, phospho-FGFR Y653/654, FGFR1, 2 (Cell Signaling, Danvers, MA, USA), KIT (Dako, Carpinteria, CA, USA), FGF-2, FRS-2, and actin (Santa Cruz Biotechnology, Santa Cruz, CA, USA). HRP-conjugated secondary antibodies for Western blotting were purchased from Santa Cruz. Primary antibodies used for IHC-staining were as follows: cleaved form of caspase-3 (Cell Signaling, Danvers, MA, USA) and Ki-67 (Spring Biosciences, Pleasanton, CA, USA).

### 4.3. Cell Lines and Culture Conditions

Human IM-sensitive vs. IM-resistant GIST T-1 cell lines were used in the present study. GIST T-1 was established from a metastatic plural tumor from a stomach GIST and contained a heterozygous 57-base pair deletion (V570-Y578) in the KIT exon 11 [39]. IM-resistant GIST T-1R subline was established in our laboratory after a continuous induction from 0.4 nM to 1000 nM IM in a stepwise increasing concentration manner [21]. GIST T-1 and T-1R cell lines were maintained in RPMI-1640 medium supplemented with 10% fetal bovine serum (FBS), 1% l-glutamine, 50 U/mL penicillin, and 50 µg/mL streptomycin. The cells were cultured in a humidified atmosphere of 5% CO_2_ at 37 °C (LamSystems, Miass, Russia).

### 4.4. Phospho-Kinase Array

The Proteome Profiler Human Phospho-Kinase Array Kit (R&D Systems, Inc., Minneapolis, MN, USA) was used to compare protein phosphorylation between the GIST T1 vs. T-1R cell lines. Briefly, 50 μg of protein was applied for the array and captured by antibodies spotted on a nitrocellulose membrane. The levels of the phospho-protein expression were then examined with an HRP-conjugated secondary antibody followed by chemiluminescence detection. The levels of chemiluminescence were detected and analyzed using the corresponding array software.

### 4.5. Colony Formation Assay

Single cell suspension was seeded in triplicates into p100 culture dishes with a total volume of 10 mL After 24 h, DMSO, IM (1 μM), BGJ398 (1 μM), or both compounds were added to the cell culture and incubated for an additional 6 days. Similarly, the MEK inhibitor U0126 (1 μmol/L) or AKT inhibitor MK2206 (1 μmol/L) was used alone or in combination with IM. At the endpoint, the cells were fixed and stained with 0.5% crystal violet for 20 min, and then washed, dried, and photographed.

### 4.6. RNA Extraction and Real-Time Quantitative PCR

Total RNA was isolated either from GIST cells using the TRIzol reagent (cat. no. BC032; RNAiso; Invitrogen; Thermo Fisher Scientific, Inc.). RNA was extracted with phenol–chloroform. Ethanol was precipitated and resuspended in diethyl pyrocarbonate-treated H_2_O. Total RNA from GIST cells was converted into cDNA using the Moloney murine leukemia virus reverse transcriptase kit (SK021; Evrogen, Moscow, Russia). One microliter of template cDNA was used in a real-time qPCR reaction with 5 × qPCR mix-HS SYBR (PB025; Evrogen, Moscow, Russia) and 10 mmol/L each of forward and reverse primers for experimental or control genes. Real-time qPCR was carried out using the CFX96 Real-Time detection system (BioRad, Hercules, CA, USA) according to the manufacturer’s protocol. PCR analysis of target genes was carried out using the following forward (F) and reverse (R) primer pairs (Table 1). Each sample was processed in parallel with assays for glyceraldehyde-3-phosphate dehydrogenase (GAPDH) which was used as the housekeeping gene, and the absolute levels of each mRNA were normalized relative to GAPDH. Quantitative data were generated on the basis of the number of cycles needed for the amplification generated fluorescence to reach a specific threshold of detection (the *C*t value).

### 4.7. GIST Xenograft Models

Subcutaneous human tumor xenografts were generated via s.c. inoculation in flank areas of 5–8-week-old female nu/nu mice with 100 μL of 1 × 107 cells/mL GIST T-1R suspension in Dulbecco’s phosphate buffered saline. The animal experimental protocols were approved by the Committee for Ethics of Animal Experimentation, and the experiments were conducted in accordance with the Guidelines for Animal Experiments in N.N. Blokhin National Medical Research Center of Oncology. All s.c. tumors were allowed to reach a volume of ~200 mm^3^ before the randomization of mice into treatment groups and drug administration. Mice were orally administered daily either 50 μL of vehicle (negative control), IM (50 mg/kg), BGJ398 (20 mg/kg), or a combination of the drugs indicated above. The animals were randomized into groups of four animals for each treatment regimen indicated above. The tumor volume, weight, and general health of the mice were recorded. After the mice had been sacrificed, tumors were excised and subjected to a histopathologic examination. Formalin-fixed, paraffin-embedded (FFPE) tissues were sectioned at 4 μM for hematoxylin and eosin (H&E) staining. Histopathologic grading of the response to the compounds was performed and was based on the microscopic amount of necrosis, myxoid degeneration, or fibrosis, with grade 1 representing a minimal response (0–10%) and grade 4 representing a maximal response (>90%) [39]. Apoptotic cells were visualized by immunohistochemical (IHC) staining for cleaved caspase-3. Ki-67 staining was used to count the number of mitotic cells. The number of positive cells was counted per 10 high power fields (HPF) at 400-fold magnification. The images were captured using Aperio’s ScanScope XT (Vista, CA, USA).

### 4.8. Statistics

All experiments were repeated three times. The results are presented as the mean ± standard error (SE) for each group. The Kaplan–Meier test was used for the survival analysis. Differences were considered significant at *p* < 0.05.

## Figures and Tables

**Table 1:** Sequences of the primers used for the real-time polymerase chain reaction.

Gene	Forward	Reverse
*FGFR1*	AACCTGCCTTATGTCCAGATCT	AGGGGCGAGGTCATCACTGC
*FGFR2*	GGCTGCCCTACCTCAAGGTTC	AGTCTGGGGAAGCTGTAATCTC
*FGFR3*	GCACACCCTACGTTACCGTG	GCCTCGTCAGCCTCCACCAG
*FGF-2*	GCTCTTAGCAGACATTGGAAG	GTGTGTGCTAACCTTACCT
*GAPDH*	GACCACAGTCCATGCCATCA	TCCACCACCCTGTTGCTGTA
